# Pharmacokinetics of a Novel Piperaquine Dispersible Granules Formulation Under Fasting and Various Fed Conditions Versus Piperaquine Tablets When Fasted in Healthy Tanzanian Adults: A Randomized, Phase I Study

**DOI:** 10.1111/cts.70133

**Published:** 2025-02-04

**Authors:** Florence A. Milando, Said Jongo, Salim Abdulla, Gloria Nyaulingo, Anneth‐Mwasi Tumbo, Sarah Mswata, Juliether Tiago, Beatus S. Bongole, Ashura Mirambo, Kamaka Kassimu, Hussein Mbarak, Mohammed A. Rashid, Ali Hamad, Michael Mihayo, Tunu Ndanzi, Omary Zuberi, Naima Saadia, Mariam Somboka, Mariam Yamba‐Yamba, Theresia Ngonyani, Amina Nditi, Margaret Msuya, Bakari Mwalimu, Hajirani M. Msuya, Khamis Awadh, Ali Ali, Anne Claire Marrast, Denis Gossen, Alice Neequaye, Myriam El‐Gaaloul, Hanu Ramachandruni, Isabelle Borghini‐Fuhrer

**Affiliations:** ^1^ Ifakara Health Institute (IHI) Bagamoyo Tanzania; ^2^ Swiss Tropical and Public Health Institute Allschwil Switzerland; ^3^ University of Basel Basel Switzerland; ^4^ MMV Medicines for Malaria Venture Geneva Switzerland; ^5^ Mangareva SRL Kraainem Belgium

**Keywords:** malaria, pharmacokinetics, piperaquine

## Abstract

Piperaquine tetraphosphate (PQP), a long‐acting antimalarial, is being considered in a combination for chemoprevention. Dihydroartemisinin‐piperaquine tablets (hard and dispersible) approved for the treatment of acute uncomplicated malaria should be administered in a fasted state, as PQP bioavailability increases with food. A new taste‐masked dispersible granules PQP formulation aims to minimize the impact of food on drug exposure. This randomized, open label, parallel group, Phase I pilot study was conducted between 24th July 2023, and 3rd January 2024, at the Ifakara Health Institute, Bagamoyo, Tanzania in 60 healthy African adults (five cohorts of 12). Single‐dose pharmacokinetics and relative systemic exposure of the oral PQP dispersible granules formulation prototype (320 mg) was compared to the hard tablet when fasted and PQP granules in three different fed conditions. In the fasted state, the relative exposure of PQP granules versus the tablet was 73.9% (90% CI 48.3, 113.0) for C_max_ and 86.5% (68.2, 109.6) for AUC_0‐t_. Following a typical East African low‐fat meal, a standard high‐fat meal, or 250 mL whole milk, the relative exposure of PQP granules versus the fasted state was 202% (90% CI 132, 311), 275% (193, 391), and 294% (203, 425) for C_max_ and 164% (124, 217), 184% (148, 228), and 195% (147, 259) for AUC_0‐t_, respectively. Both formulations were well tolerated with one drug‐related adverse event (moderate migraine). No severe or serious adverse events or clinically relevant laboratory or electrocardiograph changes were observed. PQP dispersible granules had lower systemic exposures versus the tablet when fasted, whereas various meals increased drug exposure.

**Summary**

- What is the current knowledge on the topic?
  ○ Piperaquine tetraphosphate (PQP) has potential as a component in non‐artemisinin‐based chemoprevention of malaria in Africa but should be given in the fasted state.
  ○ A novel dispersible granules formulation of PQP was developed, but systemic exposures relative to the standard hard tablet formulation and the effect of food on pharmacokinetics (PK) was unknown.
- What question did this study address?
  ○ The study evaluated the relative systemic exposures of the PQP granules and hard tablet formulations in healthy volunteers in Tanzania.
  ○ It also assessed the food effect on the PK of PQP granules when administered after low‐fat or high‐fat meals or whole milk.
- What does this study add to our knowledge?
  ○ The PQP granules formulation in the fasted state had lower exposures than the hard tablet formulation.
  ○ Administration of PQP granules in the fed state increased PQP exposures relative to the fasted state.
- How might this change clinical pharmacology or translational science?
  ○ The findings indicate that for this granules formulation of PQP, the effect of food will need to be considered in formulating new combination malaria chemoprevention therapies for use in Africa.
  ○ The study demonstrates the feasibility of conducting Phase I trials within the communities where drugs will eventually be used and using locally relevant diets to assess the effect of food on drugs.

## 1 Introduction

Malaria is a key public health issue, with an estimated 263 million cases in 2023 across 83 malaria‐endemic countries and regions [1]. The WHO African Region has the highest burden of malaria, with approximately 246 million malaria cases in 2023, representing around 94% of the global total [1].

Chemoprevention is effective in reducing the malaria burden and protecting populations most at risk, such as infants, children and pregnant women [2]. Full therapeutic courses of antimalarial medicines are administered at prescheduled times, irrespective of infection status, to treat existing infections and prevent new infections with the aim of reducing the incidence of malaria in endemic areas [2]. Currently recommended chemoprevention regimens, such as sulfadoxine‐pyrimethamine (SP) and sulfadoxine‐pyrimethamine plus amodiaquine (SPAQ), face challenges due to the increasing prevalence of *Plasmodium falciparum* harboring molecular markers of resistance to SP in Africa [3, 4]. To prepare for the potential loss of SP efficacy in chemoprevention, one approach is to combine existing antimalarials to develop new combination drugs with an appropriate risk: benefit profile to decrease morbidity and mortality in at‐risk populations.

Piperaquine tetraphosphate (PQP) is a synthetic 4‐aminoquinoline antimalarial, approved in 2011 by the European Medicine Agency as a fixed‐dose combination with dihydroartemisinin (DHA) for the treatment of uncomplicated malaria. DHA‐PQP has been investigated in chemoprevention, including intermittent treatment in pregnancy [5, 6, 7], and seasonal malaria chemoprevention (SMC) in children [8, 9, 10]. However, artemisinin derivatives are the basis of all currently available effective antimalarial treatment regimens, and partial artemisinin resistance has emerged in several African countries [1]. Thus, the need to preserve artemisinin efficacy has deterred widespread deployment of DHA‐PQP for chemoprevention.

PQP is a potential component for a chemoprevention combination drug owing to its high antimalarial efficacy and long half‐life, providing protection for up to a month following the approved 3‐day dosing regimen [11]. DHA‐PQP is currently approved as hard tablet and dispersible tablet formulations. Both formulations can be unpalatable as they have a bitter taste and PQP has low water solubility and high lipid solubility, limiting oral bioavailability [12]. PQP exposure increases by approximately three‐fold in healthy adult participants when administered with a high‐fat/high‐calorie meal [12, 13, 14], though a local low‐fat meal in South‐East Asia or administration with milk had no significant impact on PQP exposure in patients with malaria or healthy volunteers [15, 16, 17, 18]. Although a food effect can be used to boost therapeutic efficacy, such as with lumefantrine [19], PQP prolongs QT interval in a concentration‐dependent manner and the drug should be administered in the fasted state [20, 21, 22, 23]. For chemoprevention, food restriction before dosing is cumbersome, particularly for SMC where the intervention is deployed at the community level.

There is potential for re‐formulation of PQP to improve oral bioavailability, minimize the impact of food on exposure, and improve palatability [24]. A new child‐friendly, taste‐masked, PQP granules formulation was developed. It was anticipated that the smaller particle size would increase surface area, leading to faster dissolution, enhanced by the inclusion of solubilizing agents. With faster dissolution, the impact of food on absorption was expected to be attenuated and bioavailability increased. In this pilot study, the single‐dose pharmacokinetic (PK) profile of the PQP granules formulation prototype was compared to the PQP hard tablet formulation in fasting conditions (part one), and when administered with different food types (part two), to assess exposures and food effect in healthy adults. The safety, tolerability, and palatability of the granules formulation was also evaluated.

## 2 Methods

### 2.1 Ethics Statement

The study was conducted in accordance with the protocol and with the principles of the Declaration of Helsinki, the guidelines of Good Clinical Practice, and local law and regulatory authority requirements. The study protocol was approved by the National Health Research Ethics Committee (NaTHREC) in Tanzania (NIMR/HQ/R.8a/Vol.IX/4267). All participants provided written informed consent.

### 2.2 Investigational Compounds

PQP hard tablets (320 mg) and dispersible granules (26.67% w/w) were manufactured by Piramal Pharma Ltd. (Ahmedabad, India). The dispersible granules were formulated to improve PQP solubility.

### 2.3 Design and Intervention

This randomized, open label, phase 1 study was conducted at a single site (Bagamoyo Clinical Trial Facility, Ifakara Health Institute, Bagamoyo, Tanzania) between 24th July 2023 and 3rd January 2024. The study is registered at ClinicalTrials.gov (NCT05930782), and the Tanzania Clinical Trial Register (TRC‐WEB0023/CTR‐REG/0018).

Drugs were administered orally as a single dose. In part one, 24 participants were randomized (1:1) to receive either the PQP tablet (320 mg) or PQP dispersible granules (320 mg) formulation after fasting for at least 10 h. The tablet formulation was administered with 240 mL water. For the granule formulation, 1.2 g of PQP granules were weighed and dispersed in 25 mL of water. Progression to part two was contingent on review of the safety data up to Day 15 and PK data up to Day 8. In part two, after fasting for at least 10 h, 36 participants were randomized (1:1:1) to a low‐fat meal representative of the East African diet (429 Kcal, 13 g fat), a standard high‐fat meal (1019 Kcal, 62 g fat), or 250 mL cow's whole milk (150 Kcal, 8.25 g fat) (appendix Table S1). The meal had to be entirely consumed within 0.5 h, after which time PQP was administered. In parts one and two, participants did not consume food for at least 4 h after dosing. Afterwards, standardized meals were served at similar times of the day when in the unit. A palatability/tasting questionnaire using a facial hedonic scale and a Likert scale, assessing smell, sweetness, bitterness, flavor, mouthfeel/texture, aftertaste, overall liking and amount of medicine, was presented to each participant immediately after dosing, to be completed within 10 min (Table S2).

### 2.4 Sample Size and Randomization

Randomization was done using a computer‐generated randomization schedule.

This was an exploratory trial to evaluate PK and safety of each treatment group and the sample size was not based on formal statistical power. The number assigned to each treatment arm (*n* = 12) was considered adequate to assess the trial objectives, being similar to previous studies of piperaquine pharmacokinetics [12, 14, 18].

### 2.5 Participants

Participants were recruited from Bagamoyo district. Eligible participants were healthy males or females, including women of childbearing potential, aged ≥ 18 to ≤ 55 years, with a bodyweight ≥ 50 kg, and body mass index between 18.0 and 35.0 kg/m^2^. Female participants of childbearing potential were required to use highly effective contraception. Key exclusion criteria were pregnancy or lactation (or a female partner who was pregnant or lactating), known or suspected intolerance or hypersensitivity to PQP, recent or concomitant medications that may affect the study findings, clinically relevant laboratory or electrocardiogram (ECG) findings, or any disease or disorder that the investigator judged was likely to interfere with the trial or pose a risk to the participant.

### 2.6 Procedures

Participant screening was completed between Days −15 to −1, with admission on Day −1. Screening procedures included physical examination, medical history, demographic information, vital signs, a single 12‐lead ECG, biochemistry, hematology, coagulation and urinalysis, serology for HIV and hepatitis B and C, a thick blood smear to exclude malaria, urine test for drugs of abuse and breath test for alcohol, and a serum pregnancy test or follicle stimulating hormone test where appropriate. Each participant received a single dose of PQP on Day 1 and was discharged from the unit on Day 3. Thereafter, participants returned to the unit on Days 5, 8, 15, 22, and 30 (end‐of‐study visit).

### 2.7 PK Analysis

PK sampling (2 mL blood) was done at 0.5 h pre‐dose, every hour post‐dose until 8 h, then at 12, 24, 48, 120, 192, 360, 528, and 720 h post‐dose to reflect the 30‐day dosing interval in chemoprevention. Plasma samples were analyzed by Swiss BioQuant (Reinach, Switzerland) using high‐performance liquid chromatography–tandem mass spectrometry and validated methods. The lower limit of quantification for piperaquine and the piperaquine N‐oxide metabolite was 1.00 ng/mL. The calibration curves covered the concentration ranges 1.00–500 ng/mL for both analytes. Plasma concentration–time profiles for piperaquine and the N‐oxide were generated for each subject. PK parameters were estimated using non‐compartmental analysis with Phoenix WinNonlin software (v8.4 Certara USA Inc., USA).

### 2.8 Safety Assessment

Adverse events were recorded continuously from when the consent form was signed until the end‐of‐study visit. Vital signs, medical history updates, and physical examination were monitored throughout the stay within the unit and at all follow‐up visits. Samples for biochemistry assessments were taken at 48 h post‐dose and at Days 15 and 30, and hematology was assessed at Days 15 and 30. On Day 30, a thick blood smear was done to confirm the absence of malaria and pregnancy testing was done. Triplicate 12‐lead ECGs were done at 0.5 h pre‐dose and at 2, 4, 5, and 6 h post‐dose and single‐lead ECGs at 8, 12, 24, and 48 h post‐dose as well as all follow‐up visits.

Safety assessments included adverse events, defined according to the Medical Dictionary for Regulatory Activities (MedDRA version 26.0). The severity of adverse events was graded according to the WHO system [25]. ECGs were assessed for immediate safety management of the participant by the investigator, and were also over‐read at a central facility (Cardiabase, Banook, France).

### 2.9 Statistical Analysis

The safety analysis set consisted of all randomized participants who received at least one dose of PQP granules or tablet. The PK analysis set consisted of those participants in the safety set who provided sufficient blood samples for calculation of at least one PK variable.

The primary endpoint was the relative exposure based on the geometric means for the PQP granules (test) relative to the PQP tablet (reference) in the fasted state for the piperaquine area under the concentration–time curve (AUC) from time zero until 24 h (AUC_0‐24_), from time zero to 72 h (AUC_0‐72_), from time zero until the last detectable plasma concentration (AUC_0‐t_), from time zero until 168 h (AUC_0‐168_), from time zero extrapolated to infinity (AUC_0‐inf_), and the maximum plasma concentration (C_max_). The 90% confidence interval (CI) for geometric least square mean ratios of natural log‐transformed parameters were estimated for each formulation and meal conditions.

Secondary endpoints were the relative exposure of the PQP granules between the fed (test), that is, low‐fat meal, high‐fat meal, and whole milk, and fasted (reference) states, calculated and evaluated as above. Additional secondary endpoints were piperaquine PK parameters following administration of the PQP tablet (fasted) and PQP granules formulation (fasted and fed conditions), that is, C_max_, AUC_0‐72_, AUC_0‐t_, AUC_0‐168_, AUC_0‐ inf_, time to maximum plasma concentration (T_max_), terminal elimination rate constant (λ_z_), terminal elimination half‐life (t_1/2_), apparent volume of distribution during the terminal phase (V_z_/F), apparent total plasma clearance (Cl/F), and percentage of AUC due to extrapolation from the last detectable plasma concentration to infinity (%AUC_extrap_). An exploratory endpoint was the PK of the piperaquine N‐oxide metabolite in all study conditions.

Safety endpoints were the occurrence of adverse events and serious adverse events as well as clinically relevant abnormal findings from physical examination, vital signs, clinical laboratory safety parameters, and 12‐lead ECGs. Participant disposition and demographics and all safety data were presented using descriptive statistics. Categorical ECG analysis was done using Fridericia‐corrected QTc interval (QTcF), including the change from baseline. Palatability was assessed using descriptive statistics.

## 3 Results

### 3.1 Participants

**FIGURE 1:**
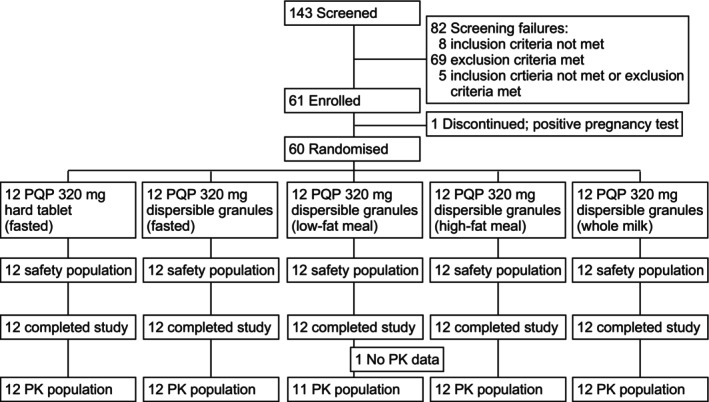
Participant disposition.

All 24 randomized participants in part one completed the study and were included in the safety and PK analysis (Figure 1). In Part 2, all 36 participants completed the study and were included in the safety analysis. One participant in the low‐fat meal group was excluded from the PK analysis because of drug exposures below the lower limit of quantification (Figure 1). Participants were aged between 20 and 51 years, an equal number of males and females were recruited, with 26/30 women being of childbearing potential. Baseline characteristics were similar across the groups for the pharmacokinetic population (Table 1) and the safety population (Table S3).

**TABLE 1:** Participant demographic characteristics (pharmacokinetic population).

Characteristic	Part one		Part two		
	PQP tablet	PQP granules	PQP granules (fed)		
	Fasted	Fasted	Low‐fat meal	High‐fat meal	Whole milk
Number of participants	12	12	11	12	12
Mean age, years (SD) [range]	29.1 (6.24) [23, 43]	28.3 (4.74) [22, 35]	29.1 (8.35) [21, 42]	28.2 (7.02) [20, 41]	32.4 (10.14) [20, 51]
Female sex, *n* (%)	7 (58.3)	4 (33.3)	7 (63.6)	6 (50.0)	6 (50.0)
Male sex, *n* (%)	5 (41.7)	8 (66.7)	4 (36.4)	6 (50.0)	6 (50.0)
Height, cm (SD) [range]	158.5 (7.80) [147.5, 174.5]	165.0 (9.64) [146.5, 181.5]	164.5 (8.39) [153.5, 182.5]	167.5 (6.16) [159.0, 178.5]	162.01 (4.92) [153.5, 172.5]
Weight, kg (SD) [range]	55.9 (6.17) [50.0, 67.0]	58.2 (5.69) [50.0, 69.0]	62.6 (7.98) [52.1, 81.0]	65.5 (8.22) [53.0, 82.0]	59.1 (8.87) [50.0, 78.0]
BMI, kg/m^2^ (SD) [range]	22.1 (3.38) [18.1, 29.0]	21.4 (3.09) [18.2, 28.7]	23.2 (3.36) [18.6, 28.2]	23.4 (3.70) [18.2, 29.1]	22.5 (3.70) [18.3, 28.8]

*Note:* All participants were black and African. All participants received PQP 320 mg.

Abbreviations: BMI, body mass index; PQP, piperaquine tetraphosphate.

### 3.2 Piperaquine Pharmacokinetics of Tablet and Granules in Fasted Condition (Part One)

**FIGURE 2:**
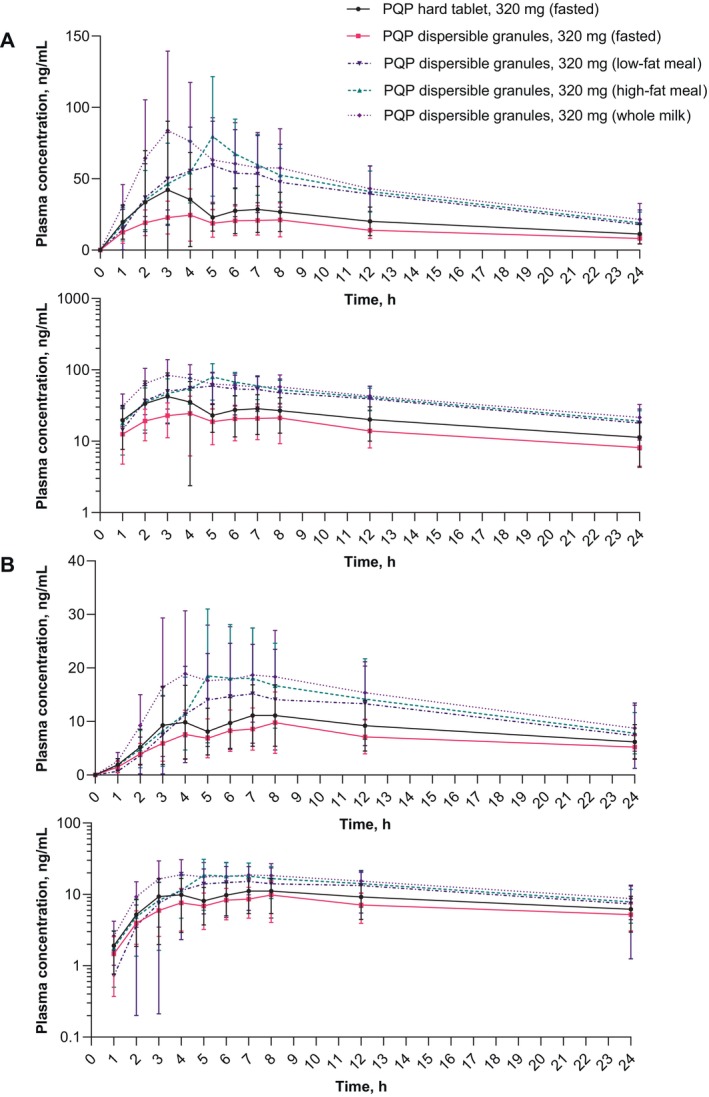
Plasma concentration–time profiles over 24 h, shown on the linear and semi‐log scales, for (A) piperaquine and (B) the piperaquine N‐oxide metabolite. Values are arithmetic mean ± standard deviation. PQP, piperaquine tetraphosphate. See appendix Figure S1 for the full profiles over 720 h.

**FIGURE 3:**
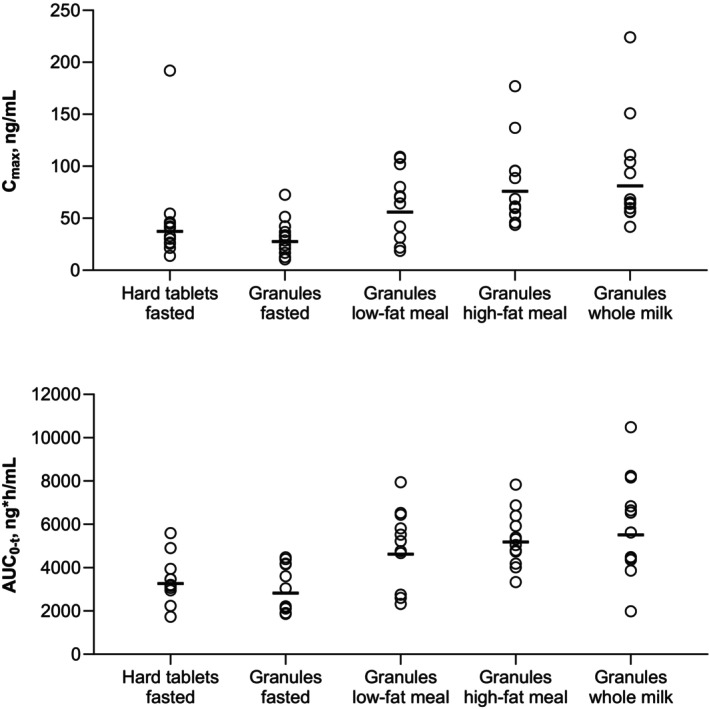
Individual participant exposure parameters for piperaquine following administration of the PQP hard tablet (320 mg) in the fasted state or PQP dispersible granules (320 mg) in the fasted state and when fed a low‐fat meal, a high‐fat meal or whole milk. Horizontal bars are the geometric mean.

Following peak piperaquine concentrations, plasma levels declined in an apparent monophasic manner for both the tablet and granules formulation (Figure 2A, Figure S1A). Maximal concentrations were observed at T_max_ between 0.980–7.03 h for the tablet and 2.05–8.00 h for the granules (Table 2). The median T_max_ was around 3 h for both formulations. The geometric mean C_max_ was 37.4 ng/mL for the tablet and 27.6 ng/mL for granules with moderate to high interparticipant variability in both groups (> 60%) (Figure 3). Exposures based on geometric AUC were higher for the tablet versus the granules with an AUC_0‐t_ of 3270 and 2820 ng.h/mL, respectively (Table 2, Figure 3). The evaluation of relative exposure between the two formulations showed that the exposure comparisons were outside of the acceptance range (80%–125%) and could not be considered equivalent. C_max_ for the granules was 73.9% (90% CI 48.3, 113.0) that of the tablet, and AUC_0‐t_ was 86.5% (90% CI 68.2, 109.6) (Table 3). The difference in exposure suggests that with the current granules formulation a higher dose would be needed to achieve the same exposures as obtained with the tablet.

**TABLE 2:** Piperaquine pharmacokinetic parameters following a 320 mg oral dose.

Parameters	PQP tablet (fasted)	PQP dispersible granules (fasted)	PQP dispersible granules (fed)		
			Low‐fat meal	High‐fat meal	Whole milk
C_max_, ng/mL	37.4 (71.5)	27.6 (61.6)	55.9 (70.2)^d^	75.9 (44.6)	81.1 (51.0)
T_max_, h	3.02 (0.980, 7.03)	3.03 (2.05, 8.00)	5.05 (3.02, 7.02)^d^	5.03 (5.02, 8.07)	4.07 (2.07, 7.03)
t_1/2_, h	309 (36.1)^a^	418 (65.1)^a^	271 (68.0)^e^	231 (31.5)^g^	424 (219.4)^g^
AUC_0‐inf_, h*ng/mL	4598 (29.3)^b^	ND^c^	4805 (47.5)^d^	6703 (18.5)^e^	5581 (19.0)^f^
AUC_0‐t_, h*ng/mL	3267 (31.7)	2824 (37.8)	4628 (43.0)^d^	5183 (24.4)	5511 (46.6)
AUC_0‐24_, h*ng/mL	450 (50.5)	324 (46.5)	740 (71.3)^d^	899 (28.4)	984 (39.6)
AUC_0‐72_, h*ng/mL	809 (42.8)	611 (43.4)	1404 (55.7)^d^	1594 (26.4)	1641 (43.8)
AUC_0‐168_, h*ng/mL	1373 (36.5)	1067 (42.2)	2323 (48.6)^f^	2642 (23.6)	2625 (44.4)
%AUC_extrap_, %	14.4 (14.8)^b^	ND^c^	10.0 (54.9)^d^	10.4 (43.6)^e^	11.8 (37.3)^f^
CL/F, L/h	69.6 (29.3)^b^	ND^c^	66.6 (47.5)^d^	47.7 (18.5)^e^	57.3 (19.0)^f^
λ_z_, 1/h	0.0022 (36.1)^a^	0.0017 (65.1)^f^	0.0026 (68.0)^f^	0.0030 (31.5)^g^	0.0016 (219.4)^g^
V_z_/F, L	23,953 (48.3)^b^	ND^c^	21,106 (98.8)^f^	14,722 (26.9)^e^	18,890 (33.2)^f^

*Note:* Values are geometric mean (geometric mean coefficient of variation [CV%]) except for T_max_ which is median (range).

Abbreviations: %AUC_extrap_, percentage of AUC due to extrapolation (i.e., AUC_t‐inf_/AUC_0‐inf_); AUC, area under the blood concentration–time curve; AUC_0‐168_, AUC from time 0 to 168 h post‐dose; AUC_0‐24_, AUC from time 0 to 24 h post‐dose; AUC_0‐72_ from time 0 to 72 h post‐dose; AUC_0‐inf_, AUC from time 0 extrapolated to infinity; AUC_0‐t_, AUC from time 0 to last detectable blood concentration; CL/F apparent total plasma clearance; C_max_, maximum observed blood concentration; ND, not determined; PQP, piperaquine tetraphosphate; t_1/2_, terminal elimination half‐life; T_max_, time to reach maximum blood concentration; V_z_/F apparent volume of distribution during the terminal phase; λ_z_, terminal elimination rate constant.

^a^ *N* = 4.

^b^ *N* = 2.

^c^ *N* = 1.

^d^ *N* = 11.

^e^ *N* = 6.

^f^ *N* = 5.

^g^ *N* = 7. All other values are *N* = 12.

**TABLE 3:** Relative exposures between PQP granules and tablet in the fasted state.

Parameter	Ratio, %	90% CI	CV%
C_max_	73.9	48.3, 113.0	66.6
AUC_0‐inf_	55.2	6.03, 506.4	29.3
AUC_0‐t_	86.5	68.2, 109.6	34.9
AUC_0‐72_	75.4	56.5, 100.7	43.1
AUC_0‐168_	77.7	59.5, 101.4	39.4

*Note:* Comparison is for piperaquine tetraphosphate (PQP) granules (*N* = 12) versus PQP tablet (*N* = 12) both in the fasted state.

Abbreviations: AUC, area under the blood concentration–time curve; AUC_0‐168_, AUC from time 0 to 168 h post‐dose; AUC_0‐72_ from time 0 to 72 h post‐dose; AUC_0‐inf_, AUC from time 0 extrapolated to infinity; AUC_0‐t_, AUC from time 0 to last detectable blood concentration; C_max_, maximum observed blood concentration; CV%, geometric mean coefficient of variation.

Peak plasma concentrations of the piperaquine N‐oxide metabolite declined in a multiphasic manner (Figure 2B, Figure S1B) from a geometric mean C_max_ of 11.1 ng/mL for the tablet and 9.74 ng/mL for granules with a median T_max_ of around 8 h for both formulations (Table S4). The geometric mean AUC_0‐t_ was 1530 and 1270 ng.h/mL for the tablet and granules, respectively (Table S4).

### 3.3 Piperaquine Pharmacokinetics of Granules in Various Fed Conditions (Part Two)

In the fed states, median T_max_ was nominally 5 h, 5 h, and 4 h, for the low‐fat, high‐fat, and whole milk groups, respectively. Following peak piperaquine concentrations, plasma levels declined in a multiphasic manner in all groups (Figure 2A). Geometric mean C_max_ values were 55.9 ng/mL (low‐fat), 75.9 ng/mL (high‐fat), and 81.1 ng/mL (whole milk) and the geometric mean AUC_0‐t_ values were 4630, 5180, and 5510 ng.h/mL, respectively (Table 2, Figure 3). Food increased C_max_ relative to the fasted state, with whole milk having the most pronounced effect (294% [90% CI 203, 425]), though similar to the high‐fat meal (275% [193, 391]), followed by the low‐fat meal (202% [90% CI 132, 311]), with a similar trend for AUC_0‐t_ (Table 4).

**TABLE 4:** Effect of food on relative exposures of PQP granules compared to the fasted state.

Parameter	PQP granules (low‐fat meal) (*N* = 11)			PQP granules (high‐fat meal) (*N* = 12)			PQP granules (whole milk) (*N* = 12)		
	Ratio, %	90% CI	CV%	Ratio, %	90% CI	CV%	Ratio, %	90% CI	CV%
C_max_	202.2	131.5, 311.1	65.8	274.8	193.3, 390.7	53.5	293.7	203.2, 424.7	56.4
AUC_0‐inf_	189.2	66.0, 542.4	47.5	263.9	177.1, 393.4	18.5	219.8	141.5, 341.3	19.0
AUC_0‐t_	163.9	124.0, 216.6	40.3	183.5	147.7, 228.0	31.7	195.2	146.8, 259.4	42.4
AUC_0‐72_	229.9	164.3, 321.8	49.5	261.0	204.8, 332.8	35.7	268.8	200.6, 360.0	43.6
AUC_0‐168_	217.8	159.7, 297.1	45.3	247.8	196.5, 312.3	34.0	246.2	184.1, 329.2	43.3

*Note:* Comparison is for piperaquine tetraphosphate (PQP) granules (N = 12) in the fasted state versus PQP granules in the fed state.

Abbreviations: AUC, area under the blood concentration–time curve; AUC_0‐168_, AUC from time 0 to 168 h post‐dose; AUC_0‐72_ from time 0 to 72 h post‐dose; AUC_0‐inf_, AUC from time 0 extrapolated to infinity; AUC_0‐t_, AUC from time 0 to last detectable blood concentration; C_max_, maximum observed blood concentration; CV%, geometric mean coefficient of variation.

Piperaquine N‐oxide plasma concentrations declined in a monophasic manner (Figure 2B). The median T_max_ was between 6 and 7 h in the three groups (Table S4). The geometric mean C_max_ values were 13.3 ng/mL (low‐fat), 18.2 ng/mL (high‐fat), and 20.0 ng/mL (whole milk), and the AUC_0‐t_ were 1600, 1790, and 2070 ng.h/mL, respectively (Table S4).

### 3.4 Safety

In part one, two participants experienced treatment emergent adverse events; one case of heart rate increased in the tablet group (103 beats/min 6 h post‐dose on Day 1) and one of upper respiratory tract infection in the granules group 8 days after dosing. Both adverse events were mild in severity and were not considered treatment related. In part two, there was one case of migraine of moderate severity in the low‐fat meal group, which was considered treatment related. This event started 4 h post‐dose and resolved within few hours with the administration of paracetamol (1000 mg). All participants recovered. No adverse events led to withdrawal from the study and there were no severe or serious adverse events. There were no clinically relevant abnormal clinical laboratory findings in either part one or part two or clinically important changes in vital signs. In part one, one participant who received the tablet formulation had a QTcF value > 450 ms (maximum 458 ms, 2 h post‐dose, vs. 448 ms at baseline; Figure S2). There were no participants with a change in QTcF > 30 ms from baseline. In part two, there were no QTcF values > 450 ms or > 30 ms change from baseline. There were no apparent trends in QTcF either by formulation or by food conditions (Figure S2).

### 3.5 Palatability

Both formulations were acceptable to participants, based on good palatability and the amount of medicine (Table S5). Overall, the PQP granules in the fasted state were preferred to the tablets, with 11/12 (91.7%) versus 9/12 (75.0%) of participants liking the formulation extremely or moderately. A post hoc analysis showed no apparent differences in response by sex, age, or BMI (Figure S3). Palatability findings were similar for PQP granules across the fed states for all three groups (Table S5).

## 4 Discussion

The PK profile and relative exposures of piperaquine following single oral doses of a novel PQP granules formulation dispersed in water was compared to the PQP hard tablet formulation in fasting conditions. Furthermore, PQP granules formulation oral exposures when administered with different food conditions among African healthy participants were assessed. Both formulations were well tolerated, with few adverse events, consistent with previous studies in healthy participants [12, 13, 14, 15, 18]. The palatability of the granules formulation was good.

In fasting conditions, the piperaquine plasma concentration–time profiles for the two formulations were similar in shape. However, overall exposure to piperaquine was decreased with the granules versus the tablet formulation for C_max_ and AUC, though variability in the exposure parameters was unchanged. There was no discernible difference in T_max_ but the t_1/2_ for the granules appeared longer (418 h) than for the tablet formulation (309 h), though limited sampling times and variability associated with half‐life determination suggests caution when interpreting these findings. The granule formulation was expected to have higher exposures compared to the tablet but this was not observed. The reasons require further investigation, for example, one possibility is that the solubilizing agents in the formulation were insufficient.

The shape of the piperaquine plasma concentration–time profiles for the three fed conditions suggested that food increased the absorption of the PQP granules compared to the fasted state. Whole milk resulted in the greatest effect, with a 2.9‐fold increase in C_max_ and 2‐fold increase in AUC_0‐t_, whereas the least effect was with the low‐fat meal with a 2‐fold and 1.6‐fold increase, respectively. Between participant variability for C_max_ was highest in the low‐fat meal group (70.2%) and lowest with the high‐fat meal group (44.6%). T_max_ was shifted to between 4 and 5 h in the fed state versus 3 h when fasted. Although there were differences in t_1/2_ between the fasted and fed states, these differences should be interpreted with caution, given the limited sampling times. The exposure of the piperaquine N‐oxide metabolite appeared to track the parent in the fed and fasted states.

The findings suggest that food slightly decreased the rate but increased the extent of absorption of PQP granules. PQP is lipophilic and administration with fat would be expected to result in increased absorption [26]. However, the greatest positive food effect was observed with whole milk, and this had the lowest fat content of the three meals. Thus, the increased absorption in the fed state does not appear to be driven by fat content alone. However, the differences were small with considerable interpatient variability (Figure 3).

In contrast with the current findings, 200 mL of chocolate milk had a minimal effect on AUC and C_max_ in Thai adult malaria patients who received a 3‐day course of DHA‐PQP [15, 16]. Similarly, there was no significant effect of 250 mL of full‐cream flavored cow's milk (8.5 g of fat) on piperaquine exposures in Papua New Guinean children with malaria who received a full course of DHA‐PQP [17]. However, there was a trend for QTcF prolongation in the milk group at 24 h and 168 h after dosing [17]. No such trends for QTcF prolongation were evident in the current study, though exposures after the single 320 mg dose were lower.

Further investigation is needed to determine why milk increased piperaquine exposures in the current study and not in previous reports. Potential considerations are the characteristics of the PQP granules formulation, the administration of PQP rather than DHA‐PQP, and the lower dose (320 vs. 960 mg) than in the previous studies, as higher doses may have resulted in saturated solubility, overcoming any food effect with milk. Also, the different populations may be a factor, that is, healthy African adults versus Asian malaria patients. Lactase persistence in certain populations affects the availability of fatty acids which will affect drug absorption [26, 27], and malaria has an extensive impact on the gastrointestinal system, which could inhibit digestion and limit fatty acid availability [28]. Notably, similar increases in bioavailability were elicited by either milk or a full‐fat breakfast for the antimalarial drug artefenomel (OZ439), which was determined by the availability of fatty acids during digestion [29]. Milk has been shown to increase the bioavailability of the antimalarial lumefantrine and has been considered as a drug delivery system for other poorly water‐soluble drugs [26].

Studies with a high‐fat meal are required by regulators as the ‘worst case food effect’. A more normal, low‐fat meal may have no clinically relevant impact on systemic exposures, improve patient compliance, and alleviate localized gastric irritation. Given the low‐fat content of standard meals within malaria endemic regions, it was assumed that the food effect of a local meal in African participants would be minimal. Previous studies in Asians who were fed a typical local meal showed a minimal effect of food on piperaquine exposures [15, 16, 17, 18]. However, the current study in the resident population of a malaria endemic region using a low‐fat typical local East African meal showed a positive food effect for the PQP granules formulation. In terms of further development of the PQP granules formulation, these findings indicate that dose optimization studies may be required as restrictions on food intake would be impractical for chemoprevention and the choice of partner drug would need to consider any potential competing food restrictions.

This exploratory study is limited by the relatively short sampling time in respect to the known long half‐life of piperaquine, and the moderate‐to‐high between participant variability. To address the high variability between participants in piperaquine pharmacokinetics, a cross‐over design would be preferable and is the favored approach when comparing formulations or assessing the effect of food [30]. However, the piperaquine elimination half‐life (~20 days) would require at least 8 weeks between doses, requiring a much longer commitment from volunteers. As piperaquine pharmacokinetics are already well characterized, a parallel design was considered acceptable for this exploratory investigation of the new formulation. For similar reasons, the effect of food on the multiple‐dose PK of the PQP granules formulation was not evaluated. For ethical reasons the study was conducted in adults, whereas the target population for malaria chemopreventive therapies in Africa is children, infants, and pregnant women.

A key strength of the study was that it was conducted in the resident population of a malaria endemic country, with local food and milk products used to evaluate food effect. Few phase I studies have been conducted in Africa, and the capacity to perform early clinical trials in malaria endemic countries has the potential to increase the scientific relevance of the findings, given that there may be nutritional, physiological, or genetic characteristics of the local population which could affect drug PK. There may also be different perceptions in terms of palatability and acceptability of different formulations, and early feedback can be incorporated into the drug development process. Importantly, early clinical research capacity empowers endemic countries to directly contribute to the development of drugs which have the potential to benefit their populations. The collaboration between MMV Medicines for Malaria Venture and the Ifakara Health Institute supported capacity strengthening and implementation of quality management systems. This provides a solid foundation for early phase clinical studies to assess interventions for malaria and other diseases of public health significance.

In conclusion, relative to the PQP hard tablet formulation, a higher dose of the novel PQP granules formulation would be needed to obtain the same drug exposures under fasting conditions. Also, food increased oral exposures of PQP granules to an extent that administration in the fasted state may still be required to minimize the risk of QTc prolongation. As PQP would need to be deployed as a combination therapy, the impact of any food restrictions on the dosing of the partner drug would also need to be considered. This study underlines the added value of conducting phase I studies in populations in the regions where the drugs will ultimately be used, as well as highlighting the increasing capacity of African centers to lead such trials.

## Author Contributions

Florence A. Milando and Isabelle Borghini‐Fuhrer wrote the manuscript. Salim Abdulla, Said Jongo, Anneth‐Mwasi Tumbo, Beatus S. Bongole, Hussein Mbarak, Mohammed A. Rashid, Amina Nditi, Margaret Msuya, Ali Ali, Anne Claire Marrast, Denis Gossen, Alice Neequaye, Myriam El‐Gaaloul, Hanu Ramachandruni, and Isabelle Borghini‐Fuhrer designed the research. Florence A. Milando, Said Jongo, Gloria Nyaulingo, Anneth‐Mwasi Tumbo, Sarah Mswata, Juliether Tiago, Beatus S. Bongole, Ashura Mirambo, Kamaka Kassimu, Hussein Mbarak, Mohammed A. Rashid, Ali Hamad, Michael Mihayo, Tunu Ndanzi, Omary Zuberi, Naima Saadia, Mariam Somboka, Mariam Yamba‐Yamba, Tunu Ndanzi, Amina Nditi, Margaret Msuya, Bakari Mwalimu, Hajirani M. Msuya, Khamis Awadh, and Ali Ali performed the research. Florence A. Milando, Said Jongo, Ali Ali, Denis Gossen, Alice Neequaye, Myriam El‐Gaaloul, Hanu Ramachandruni, and Isabelle Borghini‐Fuhrer analyzed the data.

## Conflicts of Interest

A.C.M., A.Ne., M.E‐G., H.R. and I.B‐F are employees of MMV Medicines for Malaria Venture. D.G. is the owner and director of Mangareva SRL, which received financial support from MMV Medicines for Malaria Venture to review and interpret the study results. F.A.M., S.J., S.A., G.N., A‐M.T., S.M., J.T., B.S.B., A.M., K.K., H.M., M.A.R., A.H., M.Mi., T.N., O.Z., N.S., M.S., M.Y‐Y., T.N., A.Nd., M.Ms., B.M., H.M.M., K.A., and A.A. are employees of IHI, Bagamoyo, Tanzania, which received funding from MMV Medicines for Malaria Venture for study conduct and reporting.

## Supporting information


Table S1.

Table S2.

Table S3.

Table S4.

Table S5.

Figure S1.

Figure S2.

Figure S3.

## Data Availability

De‐identified participant data are available on reasonable request and with completion of a signed data access agreement from (https://www.mmv.org/about‐us/contact‐us) referencing this publication. Data will be available for at least 5 years from publication of this study.

## References

[1] World Health Organization , “World Malaria Report 2024,” 2024, https://www.who.int/teams/global‐malaria‐programme/reports/world‐malaria‐report‐2024.

[2] World Health Organization , “WHO Guidelines for Malaria (30 November),” 2024, https://www.who.int/publications/i/item/guidelines‐for‐malaria.

[3] R. Chaturvedi , J. Chhibber‐Goel , I. Verma , S. Gopinathan , S. Parvez , and A. Sharma , “Geographical Spread and Structural Basis of Sulfadoxine‐Pyrimethamine Drug‐Resistant Malaria Parasites,” International Journal for Parasitology 51 (2021): 505–525. 33775670 10.1016/j.ijpara.2020.12.011

[4] C. E. Eboumbou Moukoko , L. P. Kojom Foko , A. Ayina , et al., “Effectiveness of Intermittent Preventive Treatment With Sulfadoxine‐Pyrimethamine in Pregnancy: Low Coverage and High Prevalence of Plasmodium Falciparum Dhfr‐Dhps Quintuple Mutants as Major Challenges in Douala, an Urban Setting in Cameroon,” Pathogens 12 (2023): 844. 37375534 10.3390/pathogens12060844 PMC10300915

[5] E. N. Muthoka , K. Usmael , S. M. Embaye , et al., “Safety and Tolerability of Repeated Doses of Dihydroartemisinin‐Piperaquine for Intermittent Preventive Treatment of Malaria in Pregnancy: A Systematic Review and an Aggregated Data Meta‐Analysis of Randomized Controlled Trials,” Malaria Journal 22 (2023): 320. 37865784 10.1186/s12936-023-04757-2 PMC10590517

[6] S. L. Eisenberg and A. E. Krieger , “A Comprehensive Approach to Optimizing Malaria Prevention in Pregnant Women: Evaluating the Efficacy, Cost‐Effectiveness, and Resistance of IPTp‐SP and IPTp‐DP,” Global Health Action 16 (2023): 2231257. 37459385 10.1080/16549716.2023.2231257 PMC10353317

[7] R. González , T. Nhampossa , G. Mombo‐Ngoma , et al., “Safety and Efficacy of Dihydroartemisinin‐Piperaquine for Intermittent Preventive Treatment of Malaria in Pregnant Women With HIV From Gabon and Mozambique: A Randomised, Double‐Blind, Placebo‐Controlled Trial,” Lancet Infectious Diseases 24 (2024): 476. 38224706 10.1016/S1473-3099(23)00738-7

[8] R. Mwaiswelo , B. Ngasala , F. Chaky , et al., “Dihydroartemisinin‐Piperaquine Effectiveness for Seasonal Malaria Chemoprevention in Settings With Extended Seasonal Malaria Transmission in Tanzania,” Scientific Reports 14 (2024): 2143. 38273019 10.1038/s41598-024-52706-z PMC10810795

[9] K. Traore , D. Coulibaly , A. K. Kone , et al., “Randomized Field Trial to Assess the Safety and Efficacy of Dihydroartemisinin‐Piperaquine for Seasonal Malaria Chemoprevention in School‐Aged Children in Bandiagara, Mali,” Journal of Infectious Diseases 229 (2024): 189–197. 37682871 10.1093/infdis/jiad387 PMC10786242

[10] I. Zongo , P. Milligan , Y. D. Compaore , et al., “Randomized Noninferiority Trial of Dihydroartemisinin‐Piperaquine Compared With Sulfadoxine‐Pyrimethamine Plus Amodiaquine for Seasonal Malaria Chemoprevention in Burkina Faso,” Antimicrobial Agents and Chemotherapy 59 (2015): 4387–4396. 25918149 10.1128/AAC.04923-14 PMC4505196

[11] J. Gutman , S. Kovacs , G. Dorsey , A. Stergachis , and F. O. ter Kuile , “Safety, Tolerability, and Efficacy of Repeated Doses of Dihydroartemisinin‐Piperaquine for Prevention and Treatment of Malaria: A Systematic Review and Meta‐Analysis,” Lancet Infectious Diseases 17 (2017): 184–193. 27865890 10.1016/S1473-3099(16)30378-4 PMC5266794

[12] I. K. Sim , T. M. Davis , and K. F. Ilett , “Effects of a High‐Fat Meal on the Relative Oral Bioavailability of Piperaquine,” Antimicrobial Agents and Chemotherapy 49 (2005): 2407–2411. 15917540 10.1128/AAC.49.6.2407-2411.2005 PMC1140540

[13] S. E. Reuter , A. M. Evans , S. Shakib , et al., “Effect of Food on the Pharmacokinetics of Piperaquine and Dihydroartemisinin,” Clinical Drug Investigation 35 (2015): 559–567. 26293519 10.1007/s40261-015-0312-8

[14] T. C. Nguyen , N. Q. Nguyen , X. T. Nguyen , D. Bui , T. Travers , and M. D. Edstein , “Pharmacokinetics of the Antimalarial Drug Piperaquine in Healthy Vietnamese Subjects,” American Journal of Tropical Medicine and Hygiene 79 (2008): 620–623. 18840754

[15] A. Annerberg , K. M. Lwin , N. Lindegardh , et al., “A Small Amount of Fat Does Not Affect Piperaquine Exposure in Patients With Malaria,” Antimicrobial Agents and Chemotherapy 55 (2011): 3971–3976. 21709087 10.1128/AAC.00279-11 PMC3165307

[16] J. Tarning , N. Lindegardh , K. M. Lwin , et al., “Population Pharmacokinetic Assessment of the Effect of Food on Piperaquine Bioavailability in Patients With Uncomplicated Malaria,” Antimicrobial Agents and Chemotherapy 58 (2014): 2052–2058. 24449770 10.1128/AAC.02318-13 PMC4023753

[17] B. R. Moore , J. M. Benjamin , S. Salman , et al., “Effect of Coadministered Fat on the Tolerability, Safety, and Pharmacokinetic Properties of Dihydroartemisinin‐Piperaquine in Papua New Guinean Children With Uncomplicated Malaria,” Antimicrobial Agents and Chemotherapy 58 (2014): 5784–5794. 25049242 10.1128/AAC.03314-14 PMC4187992

[18] T. N. Hai , S. F. Hietala , N. van Huong , and M. Ashton , “The Influence of Food on the Pharmacokinetics of Piperaquine in Healthy Vietnamese Volunteers,” Acta Tropica 107 (2008): 145–149. 18585670 10.1016/j.actatropica.2008.05.013

[19] L. Denoeud‐Ndam , A. Dicko , E. Baudin , et al., “Efficacy of Artemether‐Lumefantrine in Relation to Drug Exposure in Children With and Without Severe Acute Malnutrition: An Open Comparative Intervention Study in Mali and Niger,” BMC Medicine 14 (2016): 167. 27776521 10.1186/s12916-016-0716-1 PMC5079061

[20] T. Wattanakul , B. Ogutu , A. M. Kabanywanyi , et al., “Pooled Multicenter Analysis of Cardiovascular Safety and Population Pharmacokinetic Properties of Piperaquine in African Patients With Uncomplicated Falciparum Malaria,” Antimicrobial Agents and Chemotherapy 64 (2020): e01848. 10.1128/AAC.01848-19 PMC7318010 32312783

[21] F. Borsini , W. Crumb , S. Pace , et al., “In Vitro Cardiovascular Effects of Dihydroartemisin‐Piperaquine Combination Compared With Other Antimalarials,” Antimicrobial Agents and Chemotherapy 56 (2012): 3261–3270. 22391528 10.1128/AAC.05688-11 PMC3370756

[22] Europeans Medicines Agency , “Assessment report: Eurartesim (EMA/739355/2011),” 2011, https://www.ema.europa.eu/en/documents/assessment‐report/eurartesim‐epar‐public‐assessment‐report_en.pdf.

[23] C. Funck‐Brentano , A. Bacchieri , G. Valentini , et al., “Effects of Dihydroartemisinin‐Piperaquine Phosphate and Artemether‐Lumefantrine on QTc Interval Prolongation,” Scientific Reports 9 (2019): 777. 30692558 10.1038/s41598-018-37112-6 PMC6349839

[24] M. S. Alqahtani , M. Kazi , M. A. Alsenaidy , and M. Z. Ahmad , “Advances in Oral Drug Delivery,” Frontiers in Pharmacology 12 (2021): 618411. 33679401 10.3389/fphar.2021.618411 PMC7933596

[25] World Health Organization , “WHO Handbook for Reporting Results of Cancer Treatment,” 1979, https://iris.who.int/handle/10665/37200.

[26] B. J. Boyd , M. Salim , A. J. Clulow , G. Ramirez , A. C. Pham , and A. Hawley , “The Impact of Digestion Is Essential to the Understanding of Milk as a Drug Delivery System for Poorly Water Soluble Drugs,” Journal of Controlled Release 292 (2018): 13–17. 30359667 10.1016/j.jconrel.2018.10.027 PMC6290171

[27] M. C. Campbell and A. Ranciaro , “Human Adaptation, Demography and Cattle Domestication: An Overview of the Complexity of Lactase Persistence in Africa,” Human Molecular Genetics 30 (2021): R98–R109. 33847744 10.1093/hmg/ddab027

[28] N. Sriboonvorakul , K. Chotivanich , U. Silachamroon , et al., “Intestinal Injury and the Gut Microbiota in Patients With Plasmodium Falciparum Malaria,” PLoS Pathogens 19 (2023): e1011661. 37856470 10.1371/journal.ppat.1011661 PMC10586672

[29] M. Salim , J. Khan , G. Ramirez , et al., “Impact of Ferroquine on the Solubilization of Artefenomel (OZ439) During In Vitro Lipolysis in Milk and Implications for Oral Combination Therapy for Malaria,” Molecular Pharmaceutics 16 (2019): 1658–1668. 30830789 10.1021/acs.molpharmaceut.8b01333 PMC6448114

[30] World Health Organization Prequalification Team , “Notes on the design of bioequivalence study: dihydroartemisinin / piperaquine tetraphosphate WHO,” 2021, https://extranet.who.int/prequal/sites/default/files/document_files/BE_dihydroartemisinin‐piperaquine_March2021.pdf.

