# Supplementary material for: Pharmacokinetics of a Novel Piperaquine Dispersible Granules Formulation Under Fasting and Various Fed Conditions Versus Piperaquine Tablets When Fasted in Healthy Tanzanian Adults: A Randomized, Phase I Study
Source: Clin Transl Sci. 2025 Feb 4;18(2):e70133. doi: 10.1111/cts.70133 (PMC11794830; doi:10.1111/cts.70133)
Supplement: Supplementary file 1 — Table S1. [file CTS-18-e70133-s001.docx]

TABLE S1. Composition of the meals in the fed state.

**High-fat meal**

| **Food item** | **Calories from carbohydrates** | **Calories from protein** | **Calories from fat** | **Fat content, g** |
| --- | --- | --- | --- | --- |
| 1 cup of whole milk (250 g) | 45 | 32 | 74.3 | 8.25 |
| Eggs fried in oil ( 120 g) | 4.8 | 56.64 | 233.28 | 25.92 |
| Slices of bread (120 g) | 249.12 | 56.64 | 32.4 | 3.6 |
| 2 tablespoons of margarine (12 g) | 0 | 0 | 87.48 | 9.72 |
| Avocado (100 g) | 34 | 8 | 132.3 | 14.7 |
| Total | 332.92 | 153.28 | 559.71 | 62.19 |
| Percentage calories | 31.8 | 14.7 | 53.5 |  |
| Total calories | 1045.96 | | |  |

**Low-fat meal**

| **Food item** | **Calories from carbohydrates** | **Calories from protein** | **Calories from fat** | **Fat content, g** |
| --- | --- | --- | --- | --- |
| 1 cup of tea with milk and sugar (200g) | 24 | 10.4 | 28.8 | 3.2 |
| Boiled egg (50 g) | 2.2 | 25.2 | 47.7 | 5.3 |
| Bread white (100 g) | 207.6 | 35.2 | 27 | 3 |
| Watermelon (50 g) | 15.2 | 1.2 | 9 | 0.48 |
| Total | 249 | 72 | 112.5 | 11.95 |
| Percentage calories | 57.4 | 16.6 | 26.0 |  |
| Total calories | 433.5 | | |  |

**Whole milk**

| **Food item** | **Calories** | **Fat content, g** |
| --- | --- | --- |
| 1 cup of whole milk (250 g) | 150 | 8.25 |
