# Supplementary material for: Pharmacokinetics of a Novel Piperaquine Dispersible Granules Formulation Under Fasting and Various Fed Conditions Versus Piperaquine Tablets When Fasted in Healthy Tanzanian Adults: A Randomized, Phase I Study
Source: Clin Transl Sci. 2025 Feb 4;18(2):e70133. doi: 10.1111/cts.70133 (PMC11794830; doi:10.1111/cts.70133)
Supplement: Supplementary file 2 — Table S2. [file CTS-18-e70133-s004.docx]

TABLE S2. Acceptability and palatability questionnaire.

The following 3 questions are about the medicine you just took. Please read each one and answer by yourself. There are no ‘right’ or ‘wrong’ answers.

| Question 1: We want to know how much you like certain aspects of the product: smell, sweetness, bitterness, flavor, mouthfeel/texture and aftertaste. | | | | | |
| --- | --- | --- | --- | --- | --- |
|  | 1  Very good | 2  Good | 3  Neither good nor bad | 4  Bad | 5  Very bad |
| Sweetness |  |  |  |  |  |
| Bitterness |  |  |  |  |  |
| Flavor |  |  |  |  |  |
| Mouthfeel/texture |  |  |  |  |  |
| Aftertaste |  |  |  |  |  |
| Question 2: All aspects considered (smell, sweetness, bitterness, flavor, mouthfeel/texture and aftertaste), how would you rate your overall liking of this product? | | | | | |
| Answer to question 2 | 1  Like extremely | 2  Like moderately | 3  Neither like nor dislike | 4  Dislike moderately | 5  Dislike extremely |
| Question 3: How was the amount of the medicine? | | | | | |
| Answer to question 3 | 1  Very convenient | 2  Convenient | 3  Manageable | 4  Much | 5  Too much |
| To be completed within 10 minutes of dosing. | | | | | |
