# Supplementary material for: Pharmacokinetics of a Novel Piperaquine Dispersible Granules Formulation Under Fasting and Various Fed Conditions Versus Piperaquine Tablets When Fasted in Healthy Tanzanian Adults: A Randomized, Phase I Study
Source: Clin Transl Sci. 2025 Feb 4;18(2):e70133. doi: 10.1111/cts.70133 (PMC11794830; doi:10.1111/cts.70133)
Supplement: Supplementary file 3 — Table S3. [file CTS-18-e70133-s007.docx]

TABLE S3. Participant demographic characteristics (safety population).

| **Characteristic** | **Part one** | | **Part two** | | |
| --- | --- | --- | --- | --- | --- |
|  | **PQP tablet** | **PQP granules** | **PQP granules (fed)** | | |
|  | **Fasted** | **Fasted** | **Low-fat meal** | **High-fat meal** | **Whole milk** |
| Number of participants | 12 | 12 | 12 | 12 | 12 |
| Mean age, years (SD) [range] | 29.1 (6.24)  [23, 43] | 28.3 (4.74)  [22, 35] | 28.6 (8.15)  [21, 42] | 28.2 (7.02)  [20, 41] | 32.4 (10.14)  [20, 51] |
| Female sex, n (%) | 7 (58.3) | 4 (33.3) | 7 (58.3) | 6 (50.0) | 6 (50.0) |
| Male sex, n (%) | 5 (41.7) | 8 (66.7) | 5 (41.7) | 6 (50.0) | 6 (50.0) |
| Height, cm (SD) [range] | 158.5 (7.80)  [147.5, 174.5] | 165.0 (9.64)  [146.5, 181.5] | 164.2 (8.06)  [153.5, 182.5] | 167.5 (6.16)  [159.0, 178.5] | 162.01 (4.92)  [153.5, 172.5] |
| Weight, kg (SD) [range] | 55.9 (6.17)  [50.0, 67.0] | 58.2 (5.69)  [50.0, 69.0] | 61.7 (8.32)  [51.0, 81.0] | 65.5 (8.22)  [53.0, 82.0] | 59.1 (8.87)  [50.0, 78.0] |
| BMI, kg/m^2^ (SD) [range] | 22.1 (3.38)  [18.1, 29.0] | 21.4 (3.09)  [18.2, 28.7] | 22.9 (3.40)  [18.6, 28.2] | 23.4 (3.70)  [18.2, 29.1] | 22.5 (3.70)  [18.3, 28.8] |

BMI, body mass index; PQP, piperaquine tetraphosphate. All participants were black and African. All participants received PQP 320 mg.
