# Supplementary material for: Pharmacokinetics of a Novel Piperaquine Dispersible Granules Formulation Under Fasting and Various Fed Conditions Versus Piperaquine Tablets When Fasted in Healthy Tanzanian Adults: A Randomized, Phase I Study
Source: Clin Transl Sci. 2025 Feb 4;18(2):e70133. doi: 10.1111/cts.70133 (PMC11794830; doi:10.1111/cts.70133)
Supplement: Supplementary file 4 — Table S4. [file CTS-18-e70133-s003.docx]

TABLE S4. Piperaquine N-oxide pharmacokinetic parameters following a 320 mg oral dose of piperaquine tetraphosphate.

| **Parameters** | **PQP tablet**  **(fasted)** | **PQP granules (fasted)** | **PQP granules (fed)** | | |
| --- | --- | --- | --- | --- | --- |
|  |  |  | **Low-fat meal** | **High-fat meal** | **Whole milk** |
| C_max_, ng/mL | 11.1 (60.9) | 9.74 (54.0) | 13.3 (84.5)^e^ | 18.2 (57.0) | 20.0 (46.1) |
| T_max_, h | 8.02 (3.02, 12.1) | 8.00 (4.07, 8.12) | 7.00 (4.03, 7.05)^e^ | 6.08 (5.02, 12.0) | 6.04 (3.03, 12.0) |
| t_1/2_, h | 289 (45.5)^a^ | 412 (78.0)^c^ | 262 (38.2)^c^ | 207 (39.6)^a^ | 260 (33.0)^g^ |
| AUC_0-inf_, h*ng/mL | 3279 (–)^b^ | –^d^ | 3760 (55.3)^f^ | 2906 (42.1)^f^ | 3867 (48.1)^f^ |
| AUC_0-t_, h*ng/mL | 1528 (58.4) | 1266 (66.9) | 1595 (92.1)^e^ | 1788 (39.2) | 2069 (44.3) |
| AUC_0-24_, h*ng/mL | 173 (52.5) | 143 (42.3) | 205 (82.9)^e^ | 256 (48.8) | 300 (39.5) |
| AUC_0-72_, h*ng/mL | 392 (45.2) | 334 (39.3) | 470 (71.6)^e^ | 534 (46.6) | 602 (37.8) |
| AUC_0-168_, h*ng/mL | 750 (40.5) | 642 (43.5) | 882 (70.5)^e^ | 960 (42.0) | 1073 (37.6) |
| %AUC_extrap_, % | 13.7 (–)^b^ | –^d^ | 9.34 (66.5)^f^ | 12.0 (8.18)^f^ | 13.4 (11.1)^f^ |
| λ_z_, 1/h | 0.0024 (45.5)^a^ | 0.0017 (78.0)^c^ | 0.0026 (38.2)^c^ | 0.0034 (0.007, 39.6)^a^ | 0.0027 (33.0)^g^ |

Values are geometric mean (geometric mean coefficient of variation [CV%]) except for T_max_ which is median (range).

^a^N=7; ^b^N=1; ^c^N=9; ^d^N=0; ^e^N=11; ^f^N=3; ^g^N=8; All other values are N=12.

PQP, piperaquine tetraphosphate; Cmax, maximum observed blood concentration; Tmax, time to reach maximum blood concentration; t_1/2_, terminal elimination half-life; AUC, area under the blood concentration–time curve; AUC_0-inf_, AUC from time 0 extrapolated to infinity; AUC_0-t_, AUC from time 0 to last detectable blood concentration; AUC_0-24_, AUC from time 0 to 24 h post-dose; AUC_0-72_ from time 0 to 72 h post-dose; AUC_0-168_, AUC from time 0 to 168 h post-dose; %AUC_extrap_, percentage of AUC due to extrapolation (i.e., AUC_t-inf_/AUC_0-inf_); λ_z_, terminal elimination rate constant.
