# Supplementary material for: Pharmacokinetics of a Novel Piperaquine Dispersible Granules Formulation Under Fasting and Various Fed Conditions Versus Piperaquine Tablets When Fasted in Healthy Tanzanian Adults: A Randomized, Phase I Study
Source: Clin Transl Sci. 2025 Feb 4;18(2):e70133. doi: 10.1111/cts.70133 (PMC11794830; doi:10.1111/cts.70133)
Supplement: Supplementary file 5 — Table S5. [file CTS-18-e70133-s005.docx]

TABLE S5. Summary of palatability questionnaire (safety population).

| **Parameters** | **Response** | **PQP tablet**  **(fasted)** | **PQP granules (fasted)** | **PQP granules (fed)** | | |
| --- | --- | --- | --- | --- | --- | --- |
|  |  |  |  | **Low-fat meal** | **High-fat meal** | **Whole milk** |
| Smell | Very good | 6 (50.0) | 5 (41.7) | 5 (41.7) | 4 (33.3) | 6 (50.0) |
|  | Good | 5 (41.7) | 6 (50.0) | 5 (41.7) | 3 (25.0) | 5 (41.7) |
|  | Neither good nor bad | 1 (8.3) | 1 (8.3) | 2 (16.7) | 5 (41.7) | 1 (8.3) |
|  | Bad | 0 | 0 | 0 | 0 | 0 |
|  | Very bad | 0 | 0 | 0 | 0 | 0 |
| Sweetness | Very good | 3 (25.0) | 5 (41.7) | 5 (41.7) | 4 (33.3) | 6 (50.0) |
|  | Good | 4 (33.3) | 4 (33.3) | 5 (41.7) | 3 (25.0) | 5 (41.7) |
|  | Neither good nor bad | 4 (33.3) | 3 (25.0) | 2 (16.7) | 5 (41.7) | 1 (8.3) |
|  | Bad | 1 (8.3) | 0 | 0 | 0 | 0 |
|  | Very bad | 0 | 0 | 0 | 0 | 0 |
| Bitterness | Very good | 4 (33.3) | 3 (25.0) | 1 (8.3) | 0 | 1 (8.3) |
|  | Good | 5 (41.7) | 3 (25.0) | 4 (33.3) | 4 (33.3) | 3 (25.0) |
|  | Neither good nor bad | 3 (25.0) | 5 (41.7) | 6 (50.0) | 7 (58.3) | 8 (66.7) |
|  | Bad | 0 | 0 | 0 | 1 (8.3) | 0 |
|  | Very bad | 0 | 0 | 1 (8.3) | 0 | 0 |
| Flavor | Very good | 3 (25.0) | 7 (58.3) | 3 (25.0) | 3 (25.0) | 2 (16.7) |
|  | Good | 5 (41.7) | 3 (25.0) | 5 (41.7) | 6 (50.0) | 7 (58.3) |
|  | Neither good nor bad | 3 (25.0) | 2 (16.7) | 3 (25.0) | 3 (25.0) | 3 (25.0) |
|  | Bad | 1 (8.3) | 0 | 0 | 0 | 0 |
|  | Very bad | 0 | 0 | 1 (8.3) | 0 | 0 |
| Mouthfeel/texture | Very good | 5 (41.7) | 7 (58.3) | 3 (25.0) | 0 | 2 (16.7) |
|  | Good | 6 (50.0) | 2 (16.7) | 4 (33.3) | 2 (16.7) | 8 (66.7) |
|  | Neither good nor bad | 1 (8.3) | 3 (25.0) | 5 (41.7) | 9 (75.0) | 2 (16.7) |
|  | Bad | 0 | 0 | 0 | 1 (8.3) | 0 |
|  | Very bad | 0 | 0 | 0 | 0 | 0 |
| Aftertaste | Very good | 3 (25.0) | 5 (41.7) | 1 (8.3) | 0 | 3 (25.0) |
|  | Good | 8 (66.7) | 4 (33.3) | 6 (50.0) | 6 (50.0) | 6 (50.0) |
|  | Neither good nor bad | 1 (8.3) | 3 (25.0) | 4 (33.3) | 3 (25.0) | 2 (16.7) |
|  | Bad | 0 | 0 | 1 (8.3) | 3 (25.0) | 1 (8.3) |
|  | Very bad | 0 | 0 | 0 | 0 | 0 |
| Overall | Like extremely | 6 (50.0) | 9 (75.0) | 5 (41.7) | 4 (33.3) | 7 (58.3) |
|  | Like moderately | 3 (25.0) | 2 (16.7) | 6 (50.0) | 7 (58.3) | 4 (33.3) |
|  | Neither like nor dislike | 3 (25.0) | 1 (8.3) | 1 (8.3) | 1 (8.3) | 1 (8.3) |
|  | Dislike moderately | 0 | 0 | 0 | 0 | 0 |
|  | Dislike extremely | 0 | 0 | 0 | 0 | 0 |
| Amount of medicine | Very convenient | 3 (25.0) | 3 (25.0) | 1 (8.3) | 3 (25.0) | 6 (50.0) |
|  | Convenient | 5 (41.7) | 8 (66.7) | 7 (58.3) | 2 (16.7) | 1 (8.3) |
|  | Manageable | 4 (33.3) | 1 (8.3) | 4 (33.3) | 6 (50.0) | 5 (41.7) |
|  | Much | 0 | 0 | 0 | 1 (8.3) | 0 |
|  | Too much | 0 | 0 | 0 | 0 | 0 |

Data are number of participants (%). PQP, piperaquine tetraphosphate.
