# Supplementary material for: Pharmacokinetics of a Novel Piperaquine Dispersible Granules Formulation Under Fasting and Various Fed Conditions Versus Piperaquine Tablets When Fasted in Healthy Tanzanian Adults: A Randomized, Phase I Study
Source: Clin Transl Sci. 2025 Feb 4;18(2):e70133. doi: 10.1111/cts.70133 (PMC11794830; doi:10.1111/cts.70133)

FIGURE S2. Post-dose individual participant Fridericia-corrected QT interval (QTcF) values. PQP, piperaquine tetraphosphate.

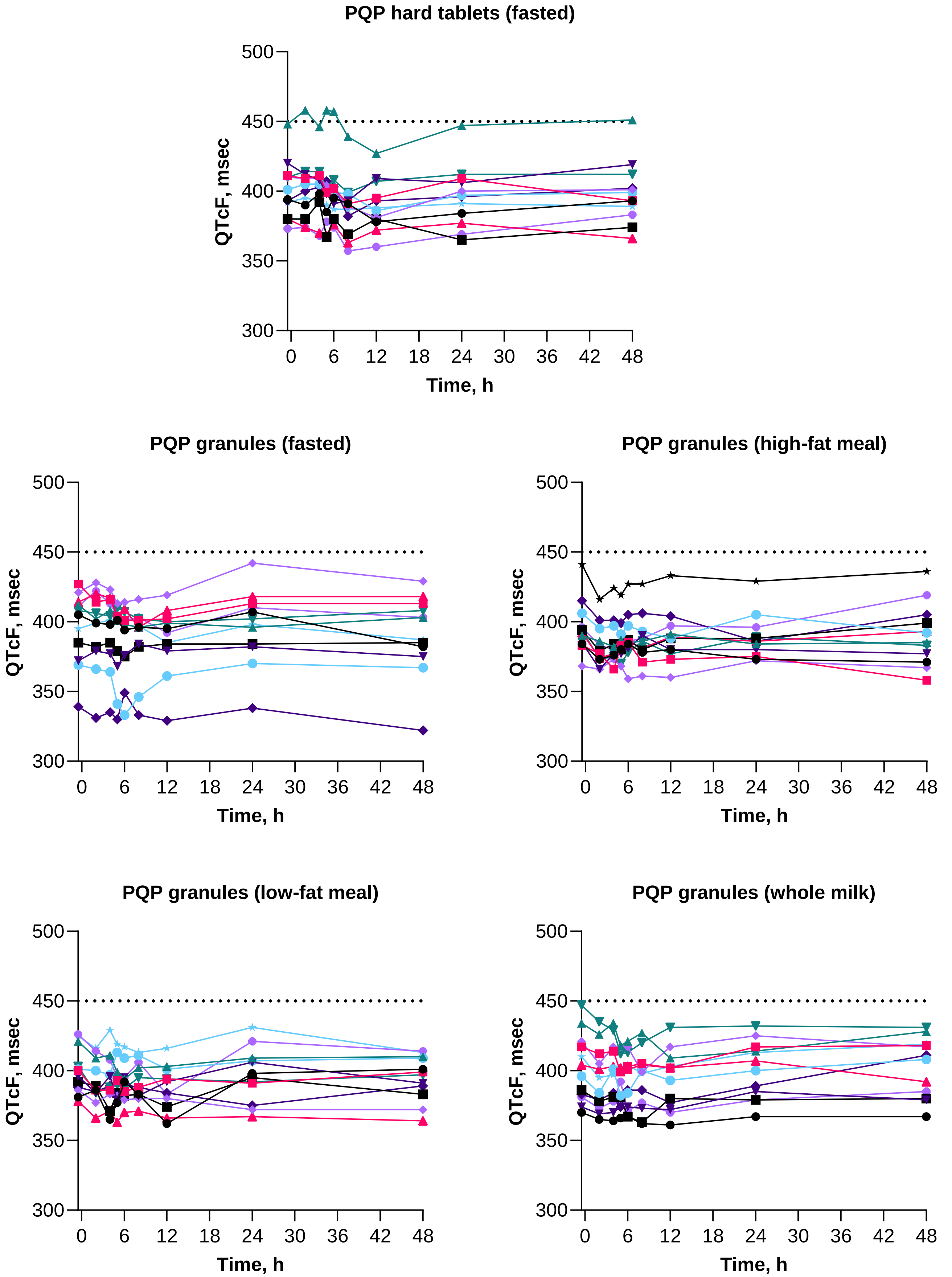

Supplement: Supplementary file 7 — Figure S2. [file CTS-18-e70133-s008.pdf]
