# Supplementary figures and images for: Pharmacokinetics of a Novel Piperaquine Dispersible Granules Formulation Under Fasting and Various Fed Conditions Versus Piperaquine Tablets When Fasted in Healthy Tanzanian Adults: A Randomized, Phase I Study
Source: Clin Transl Sci. 2025 Feb 4;18(2):e70133. doi: 10.1111/cts.70133 (PMC11794830; doi:10.1111/cts.70133)

**FIGURE S3.** Overall palatability and amount of drug by sex, age and BMI (safety population).

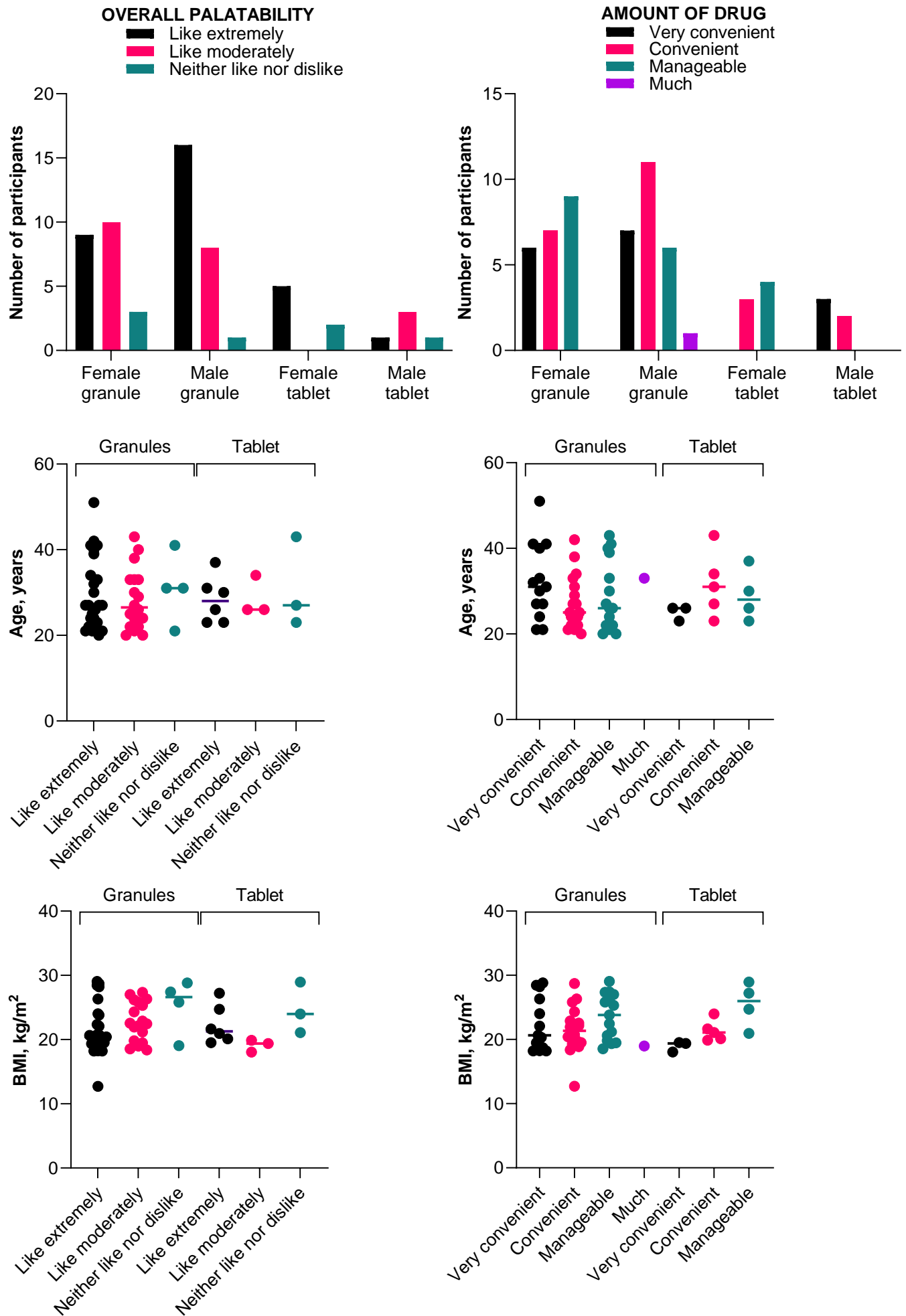

Supplement: Supplementary file 8 — Figure S3. [file CTS-18-e70133-s002.pdf]
