# Supplementary material for: Pharmacokinetics of a Novel Piperaquine Dispersible Granules Formulation Under Fasting and Various Fed Conditions Versus Piperaquine Tablets When Fasted in Healthy Tanzanian Adults: A Randomized, Phase I Study
Source: Clin Transl Sci. 2025 Feb 4;18(2):e70133. doi: 10.1111/cts.70133 (PMC11794830; doi:10.1111/cts.70133)

FIGURE S1. Full plasma–concentration–time profiles for (A) piperaquine and (B) the piperaquine N-oxide metabolite. Values are arithmetic mean ± standard deviation. PQP, piperaquine tetraphosphate.

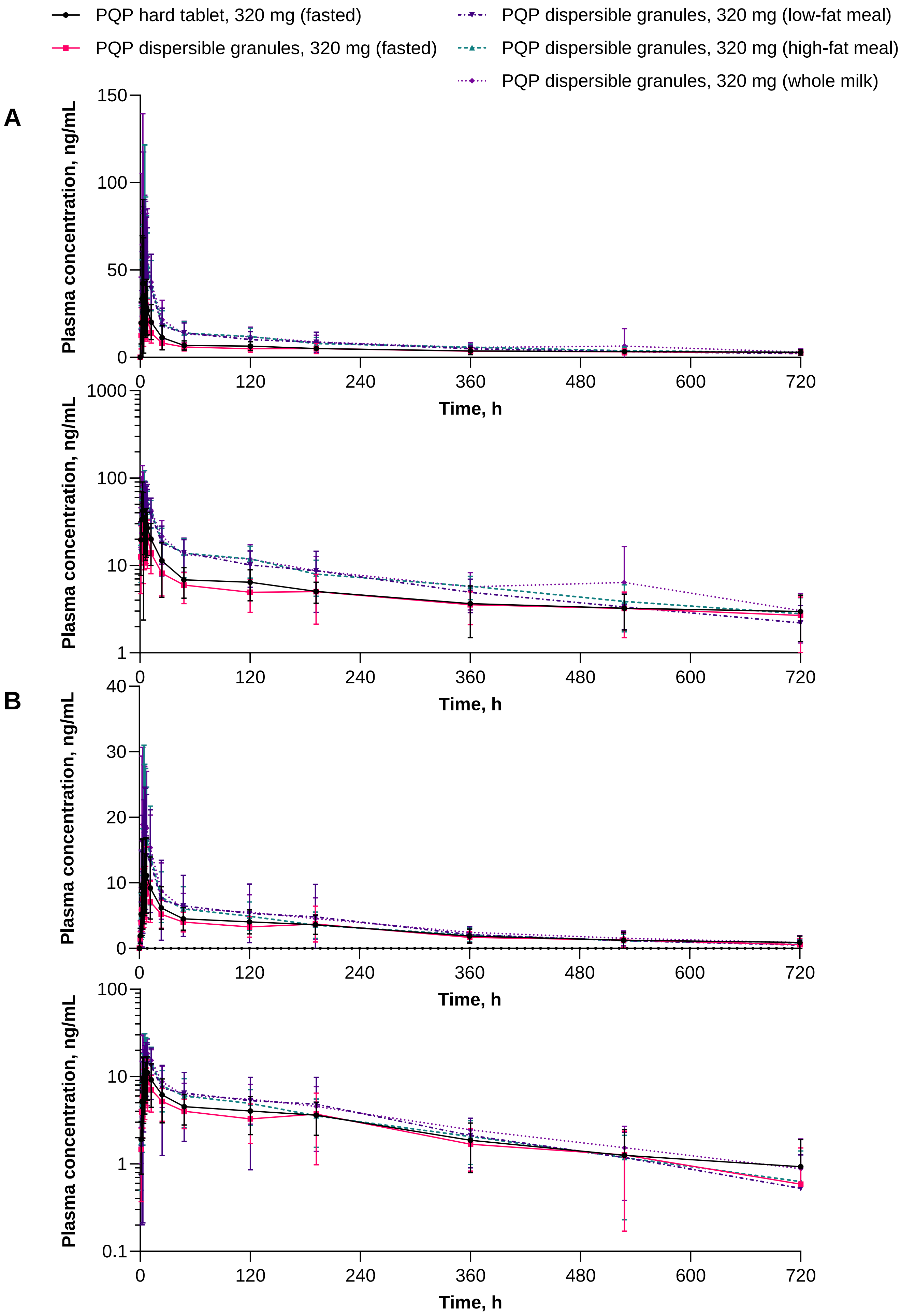

Supplement: Supplementary file 6 — Figure S1. [file CTS-18-e70133-s006.pdf]
